# Where do herbivore-induced plant volatiles go?

**DOI:** 10.3389/fpls.2013.00185

**Published:** 2013-06-11

**Authors:** Jarmo K. Holopainen, James D. Blande

**Affiliations:** Department of Environmental Science, University of Eastern FinlandKuopio, Finland

**Keywords:** terpenoids, monoterpenes, green leaf volatiles, semivolatiles, secondary aerosols

## Abstract

Herbivore induced plant volatiles (HIPVs) are specific volatile organic compounds (VOC) that a plant produces in response to herbivory. Some HIPVs are only produced after damage, while others are also produced by intact plants, but in lower quantities. Among the known functions of HIPVs are within plant volatile signaling to activate systemic plant defenses, the priming and activation of defenses in neighboring plants and the attraction of natural enemies of herbivores. When released into the atmosphere a plant's control over the produced compounds ends. However, many of the HIPVs are highly reactive with atmospheric oxidants and their atmospheric life times could be relatively short, often only a few minutes. We summarise the potential ecological and atmospheric processes that involve the reaction products of HIPVs in their gaseous, liquid and solid secondary organic aerosol (SOA) forms, both in the atmosphere and after deposition on plant surfaces. A potential negative feedback loop, based on the reactions forming SOA from HIPVs and the associated stimulation of sun screening cloud formation is presented. This hypothesis is based on recent field surveys in the geographical areas facing the greatest degree of global warming and insect outbreaks. Furthermore, we discuss how these processes could benefit the individual plant or conspecifics that originally released the HIPVs into the atmosphere. Further ecological studies should aim to elucidate the possible reasons for biosynthesis of short-lived volatile compounds to have evolved as a response to external biotic damage to plants.

## Introduction

Most vascular plants constitutively emit volatile organic compounds (VOCs), but emissions may substantially increase and diversify under conditions of abiotic and biotic stress (Holopainen and Gershenzon, 2010). Feeding by herbivores was found to induce the emission of novel volatile compounds often referred to as herbivore-induced plant volatiles (HIPVs) (Hare, 2011) that attract natural enemies of the herbivores. This was shown for the first time in seminal studies conducted with spider mites and predatory mites by Dicke and Sabelis (1988) and with moth larvae and parasitic wasps by Turlings et al. (1990). Since the first studies of plant volatiles, interest in the synthesis and control of volatiles by plants and their ecological and atmospheric functions have increased substantially (Dicke and Loreto, 2010) and several other ecophysiological and ecological effects of constitutive and inducible plant volatiles have been described (Laothawornkitkul et al., 2009; Holopainen and Gershenzon, 2010; Loreto and Schnitzler, 2010; Peñuelas and Staudt, 2010).

HIPVs are often expected to increase the fitness of the emitting plant either directly or indirectly (Dicke, 2009). Direct defense reduces herbivore approach and attack or decreases the herbivore's consumption rate, but the roles of HIPVs could be complicated. For instance, the ratios of compounds in the typical HIPV profiles of *Quercus robur* trees correlate with the tree's susceptibility to herbivore damage (Ghirardo et al., 2012). Trees representive of an herbivore-resistant phenotype emitted HIPVs that included the sesquiterpenes α-farnesene and germacrene D and were avoided by females of the defoliating moth *Tortrix viridian* (Ghirardo et al., 2012). However, in the same outbreak area trees emitting other typical HIPVs including the monoterpene β-ocimene and homoterpene (*E*)-4,8-dimethyl-1,3,7-nonatriene (DMNT) were susceptible and were largely defoliated (Ghirardo et al., 2012).

Indirect defense involves the recruitment of natural enemies of herbivores to increase predation or parasitism rates and eventually reduce damage. Many laboratory reports have given support to this hypothesis (Hare, 2011). However, there is scarce field-based -evidence that attraction of natural enemies by HIPVs actually reduces herbivore populations (Kessler and Baldwin, 2001). Furthermore, studies to show improved Darwinian fitness in plants emitting HIPVs, i.e., more offspring in the next generation, are lacking (Hare, 2011). A current ecological view of the role of HIPVs is as components of a wider infochemical web, including e.g., pollinators and root synergists, that overlay the food webs of a community rather than simply defending against attackers or attracting carnivores (Dicke and Baldwin, 2010). Ghirardo et al. (2012) concluded that for *Q. robur*, the strategy of emitting herbivore-repellent rather than natural enemy attracting HIPVs, appears to be the better mechanism for avoiding defoliation. However, when plants are influenced by a diverse community of chewing and sucking herbivores, a single HIPV compound could be an efficient repellent against one herbivore, but act as an attractant of another herbivore and many of the community's predators and parasitoids (Xiao et al., 2012).

HIPVs are not only emitted by aboveground parts of plants. Many plant species have an extensive root system where HIPV-releasing resins are stored (Kivimaenpää et al., 2012) or HIPVs synthesized (Degenhardt et al., 2009) and released in the soil air space and eventually to the atmosphere. Root feeding by herbivores induces the emission of HIPVs by the root system, which act as belowground attractants for parasitic nematodes (van Tol et al., 2001; Rasmann et al., 2005). Aboveground herbivory also has a systemic impact on belowground HIPV production and vice versa (Erb et al., 2009). Defoliation of *Pinus syvestris* by diprionid sawflies induced substantial HIPV emissions from the shoots, but resulted in significant reduction in monoterpene and sesquiterpene emissions from the root system (Ghimire et al., 2013). This was expected to be related to reduced carbon allocation to below-ground parts after defoliation. Arbuscular mycorrhizal (AM) infection of bean plant roots affected the HIPV composition emitted by foliage by making it less attractive to predators (Schausberger et al., 2012), whereas an ectomycorrhizal (EM) root symbiont did not affect the terpene pool of pine needles (Manninen et al., 1998). These studies highlight the complex and systemic nature of HIPVs and the need for a holistic view of a plant's volatile emissions and their various related roles.

HIPV compounds typically have relatively short atmospheric lifetimes after release from plants, which may limit the efficiency with which they attract natural enemies of herbivores and mediate other ecological interactions (Yuan et al., 2009). However, reactive VOCs have various functions in the atmospheric processes, such as formation of ozone in NO_x_ polluted atmospheres (Atkinson and Arey, 2003), formation of OH-radicals (Mentel et al., 2009), formation of organic nitrates (Pratt et al., 2012) and formation of secondary aerosols (SOA) (Joutsensaari et al., 2005; Kiendler-Scharr et al., 2009; Mentel et al., 2009; Virtanen et al., 2010). Laothawornkitkul et al. (2009) divided the various functions of plant VOCs into three broad categories; biological, chemical and physical.

In this review we focus on the different roles and fate of inducible VOC molecules after release from the VOC synthesizing plant including biological, chemical and physical aspects. Furthermore, we discuss why biosynthesis and emission of short-lived volatile compounds has evolved as a general response to external biotic damage to plants. We will pay attention to the biological and ecological role of VOCs post-emission and their atmospheric reaction products. These effects may take place in their gaseous, liquid and solid organic particulate forms in the atmosphere, but probably also after deposition on plant surfaces. We also discuss how the post-emission reaction products of VOCs may improve plant fitness. Finally we try to present the potential routes that the carbon fixed by a plant and bound in VOC molecules will ultimately take.

## Major groups of HIPVs

**Figure 1:**
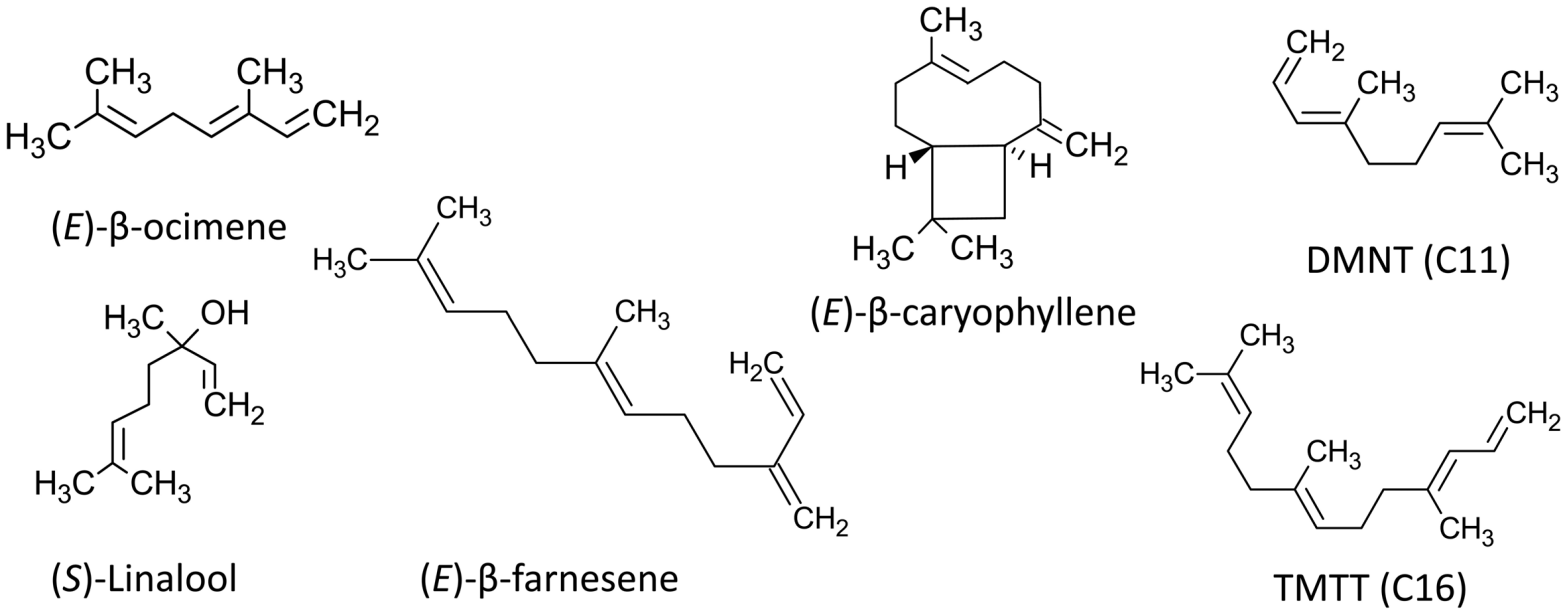
**Examples of molecular structures of isoprenoid HIPVs showing double bonds**.

The majority of the typically documented herbivore-induced plant volatiles can be classified in three major chemical groups based on their biosynthesis pathways or their known within-plant functions (Holopainen and Gershenzon, 2010). First, and the dominating group of constitutively emitted VOCs and HIPVs in many plant species, are the terpenoids, which are produced by two separate pathways, one active in plastids (MEP) and one (MVA) in the cytosol (Loreto and Schnitzler, 2010; Maffei, 2010). The volatile terpenoids (Figure 1) include the five carbon (C5) isoprene molecule and a range of molecules comprising various multiples of this basic C5 unit, including monoterpenes (C10), homoterpenes (C11 or C16) and sesquiterpenes (C15). The second group is the C6 lipoxygenase (LOX) products better known as Green Leaf Volatiles (GLVs). GLVs, such as (*Z*)-3-hexenal and (*Z*)-3-hexenyl acetate are compounds released after mechanical or other destructive damage to cell membranes (Maffei, 2010; Holopainen, 2011). The third group is the volatile aromatic compounds such as methyl salicylate and indole produced by the shikimate pathway and containing an aromatic ring (Maffei, 2010). In addition to these three main groups there are a multitude of other volatile compounds that are specific to varying degrees such as to an order, genus or species. The volatile plant hormone ethylene has often been considered an inducible volatile and has several functions in plant physiological processes and growth, while membrane-bound ethylene receptors are well known (Holopainen and Gershenzon, 2010).

HIPVs can also be classified based on their volatility or the atmospheric life times of the compounds in atmospheres with standardized levels of reactive scavengers such as ozone (O_3_), nitrate (NO_3_) or hydroxyl (OH^−^) radicals (see Holopainen, 2011; Holopainen et al., 2013 and Table 1). These scavengers appear in higher concentrations in polluted air, but they are common in ambient air and are involved in important chemical and physical processes in the atmosphere. Significantly, plant VOCs also participate in their formation (Hallquist et al., 2009). A greater number of C-C double bonds in the VOC molecules (Figure 1) will make them more prone to reactions with atmospheric radicals, degrade faster and form more particles (Hoffmann et al., 1997; Atkinson and Arey, 2003; Pinto et al., 2010).

**Table 1:** **Examples of typical herbivore induced plant volatiles (HIPV) and their estimated atmospheric life times in the detected concentrations of three major reactive air pollutants in less polluted areas**.

	**HIPV compounds and their estimated atmospheric life times**			
**Atmospheric pollutants**	<10 min	**10 min–1 h**	**1 h–24 h**	<**24 h**
Ozone (O_3_)	**β-Caryophyllene**	*cis*-/*trans*-Ocimene, Linalool, DMNT, TMTT **β-Farnesene**	α-Pinene, β-Phellandrene, Limonene	Methyl salicylate
			*cis*-3-Hexenyl acetate,	
			*cis*-3-Hexen-1-ol, *cis*-3-Hexenal^1)^	
Hydroxyl radical (OH)		*cis*-/*trans*-Ocimene, β-Phellandrene, Limonene, Linalool, **β-Caryophyllene**, DMNT, TMTT	α-Pinene,	Methyl salicylate
			*cis*-3-Hexenyl acetate,	
			*cis*-3-Hexen-1-ol	
			*cis*-3-Hexenal	
Nitrate radical (NO_3_)	α-Pinene, *cis*-/*trans*-Ocimene, β-Phellandrene, Limonene Linalool, **β-Caryophyllene** DMNT, TMTT,	**β-Farnesene** Methyl salicylate	*cis*-3-Hexenyl acetate,	
			*cis*-3-Hexen-1-ol	

Monoterpenes (normal font), sesquiterpenes (bold font), homoterpenes (capital font), GLVs (underlined) and aromatic compounds (small caps)

*Data is compiled from the following sources: Atkinson and Arey (2003), Arneth and Niinemets (2010), Roger Atkinson, personal communication., Ulo Niinemets, personal communication, Canosa-Mas et al. (2002), Holopainen et al. (2013)*.

*Pollutant concentrations used were O3: 30 ppb 24-h average, OH: 0.074 pmol mol^−1^ 12-h average, NO_3_: 9.3 pmol mol^−1^, 24-h average. OH concentrations can be measured mostly in day time and NO_3_ only in night time, due to solar UV-radiation (Holopainen et al., 2013)*.

## Succession of induced VOCs

When a plant is attacked by a herbivore, there is a succession of different inducible volatile compounds appearing in the emission bouquet. Inducible defenses of herbivore-attacked plants involve herbivore perception, transcriptional responses, protein formation and biosynthetic responses (Dicke, 2009). In plant species storing a constitutively synthesized volatile mixture, these compounds will volatilize very rapidly upon rupture of storage structures such as glandular trichomes and constitute the first response to external damage (Jansen et al., 2011). In this case the emission is the result of mechanical injury, which will occur before the attacker induces the biosynthesis of volatiles in growing plant tissues. The GLV emissions have a time lag between herbivore-feeding and compound emission of from just a few seconds to several minutes and these compounds show very rapid response to mechanical or biological damage to cell membranes. The C18 fatty acids of membranes are cleaved to C12 and C6 compounds by hydroperoxide lyases with 3-*Z*-hexenal (aldehyde) being the first C6 GLV compound synthesized by the lipoxygenase (LOX)/lyase pathway (Maffei, 2010). This compound is then converted to other common C6 GLVs such as (*E*)-2-hexenal (aldehyde), 3-hexenol (alcohol) and (*Z*)-3-hexenyl acetate (ester) (Shiojiri et al., 2006). Geometrid moth feeding induces emissions of GLVs, which peak soon after larval feeding starts on deciduous tree foliage (Blande et al., 2007), while on-line monitoring of HIPVs shows that GLV peaks can even reveal the timing of larval feeding periods (Schaub et al., 2010).

Continuous mechanical injury (Mithofer et al., 2005), oral secretions of herbivores (Turlings et al., 1990), plant cell membrane damage by biotrophic fungal leaf pathogens (Toome et al., 2010; Jansen et al., 2011) or bacterial pathogens (Yi et al., 2009) elicit signal transduction pathways that are mediated by phytohormones such as jasmonic acid (JA) and ethylene in the case of chewing herbivores, salicylic acid (SA) and ethylene in the case of fungal pathogens and result in the synthesis of typical HIPV terpenoids and aromatic compounds (Jansen et al., 2011). There is variability in the succession of different herbivore-induced terpenoid emissions, which could be partly due to variable allocation of precursors into the different biosynthesis pathways. Biosynthesis of the homoterpene (*E*)-DMNT, for example, could originate predominantly from the MVA-pathway in herbivore-stressed plants, while the fungal elicitor alamethicin stimulates the biosynthesis of (*E*)-DMNT via the MEP-pathway (Bartram et al., 2006). In *Alnus* foliage damaged by geometrid moths, emissions of (*E*)-β-ocimene and (*E*)-DMNT peaked on day 3 (Copolovici et al., 2011). In the same study the emission kinetics of the sesquiterpene (*E*,*E*)-α-farnesene tended to be biphasic with peaks on days 2 and 4 after the start of larval feeding. Emission rates of the induced LOX products, (*E*)-β-ocimene and (*E*,*E*)-α-farnesene were positively correlated with the number of larvae feeding (Copolovici et al., 2011).

Variation in the feeding strategies of herbivores can result in profound variation in the volatiles emitted by damaged plants. Herbivores that feed via stylets, such as aphids, inflict apparently minor mechanical damage, but still induce the emission of a rich blend of volatiles including both GLVs and terpenoids (Gosset et al., 2009). Sustained feeding by aphids and colony growth can also result in large increases in emission of methyl salicylate (Blande et al., 2010), which can take several days to start appearing in emission bouquets. The volatiles induced by chewing herbivores can vary with the life stage of the herbivore, with early instars (first to fourth) of *Pseudaletia separata* larvae inducing different volatile bouquets to larger more advanced larvae (fifth to sixth instars) (Takabayashi et al., 1995). In this case, foraging parasitoids are able to distinguish between the volatile blends induced by potential host larvae (the younger instars) and larvae that are too old to be used as hosts and may actually constitute a threat through their aggressive defensive behaviors (Takabayashi et al., 1995). In an alternative system, with *Pieris brassicae* feeding on Brussels sprouts, *Cotesia glomerata* parasitoids do not appear to determine larval instar through volatile emissions, but can determine presence of suitable hosts through other cues on infested leaves, without necessarily contacting the host itself (Mattiacci and Dicke, 1995). Elicitors in the saliva of the herbivores are responsible for alterations in the herbivore-induced blend, the specificity and range of which can vary (Mattiacci and Dicke, 1995; Takabayashi et al., 1995; Roda et al., 2004). It has also been shown that deposition of eggs by the Brassica specialising Lepidopteran *Pieris brassicae* can induce changes in the expression of hundreds of genes (Little et al., 2007; Fatouros et al., 2008) and emission of volatiles that are attractive to its parasitic wasps (Fatouros et al., 2012).

In *Salix* hybrid plantlets infected with *Melampsora epitea* leaf rust fungi, the total monoterpene emissions did not change although a stress-signaling compound (*Z*)-β-ocimene showed an increase in infected plants on several days. The infection also increased the emission of sesquiterpenes and LOX products by factors of 175-fold and 10-fold, respectively (Toome et al., 2010). The induced VOCs showed two clear peaks during the experiment; at 6–7 and 12 days post-infection, whereby the relative volatile emission signal increased to about 6-fold that of uninfected plants. Peak emission periods were directly connected to rust infection with day 6 corresponding with the appearance of the first rust pustules on the leaves and day 12 corresponding with necrosis developing around several pustules (Toome et al., 2010).

Isoprene -a major biogenic VOC released from vegetation—and some monoterpenes are constitutively emitted, but are induced by elevated temperatures, which can greatly enhance the overall emission of these compounds (Loreto and Schnitzler, 2010). Of these compounds, isoprene in particular is not induced by fungal pathogens or insect feeding. Toome et al. (2010) reported that isoprene emissions from *Salix* hybrids with rust-infected leaves decreased 3-fold compared to controls, Ghirardo et al. (2012) found that *Tortrix viridiana* larval feeding did not affect isoprene emissions from *Quercus robur* and Blande et al. (2007) found that emission of isoprene in *Populus* hybrids did not respond significantly to geometrid moth or leaf weevil feeding.

Several studies have implicated the blend of volatiles emitted by plants either constitutively, or after herbivore-damage, to play an important role in the behavior of foraging insects. Aphids in particular have been shown to utilize blends of volatiles emitted by undamaged plants as host location cues (Bruce and Pickett, 2011; Webster, 2012), while parasitoids have also been shown to utilize chemical blends to locate their hosts (Pareja et al., 2009; Clavijo McCormick et al., 2012). It is clear that the blend emitted by plants can evolve as the degree of herbivore-induced stress changes. Differences in degradation rates of certain chemicals could result in rapid changes to the blend, by reducing the proportions of compounds relative to each other (Pinto et al., 2007a), which could render the blend less effective as a cue for foraging insects (Pinto et al., 2007b).

## Known functions and effects of HIPVs

### HIPVs and plant adaptation to abiotic stresses

Emissions of plant volatiles are strongly dependent on physical conditions and the changes in these conditions could rapidly “induce” emissions or alter emission dynamics. Ambient temperature and light conditions affect synthesis and emissions of terpenoids particularly strongly (Niinemets et al., 2004). Emissions of many HIPVs are also induced by a range of abiotic factors such as drought, CO_2_ level and ozone (Vuorinen et al., 2004; Dicke and Loreto, 2010; Peñuelas and Staudt, 2010). However, inducibility of HIPVs can be affected by environmental conditions during attack by herbivores (Gouinguene and Turlings, 2002; Holopainen and Gershenzon, 2010). In plant leaves isoprene and monoterpenes have been shown to protect the photosynthetic apparatus of plants from damage under high temperature episodes and maintain the photosynthetic capacity under temperature increase (Behnke et al., 2007; Loreto and Schnitzler, 2010).

### Within plant signals

If HIPVs are considered to improve the fitness of a unitary plant, their role in signaling between vascularly separate parts of an individual could be one of their primary functions (Karban et al., 2006; Frost et al., 2007; Rodriguez-Saona et al., 2009; Shiojiri et al., 2009). Such observations, whereby volatile signaling between herbivore-damaged and intact branches results in unwounded branches being better protected against subsequent herbivore-attack, have been made in several plant species, including lima bean, blueberry, sagebrush and hybrid poplar (Karban et al., 2006; Frost et al., 2007; Heil and Silva Bueno, 2007; Rodriguez-Saona et al., 2009). The relevant signals might be mixtures of HIPVs (Rodriguez-Saona et al., 2009) or single compounds such as the GLV (*Z*)-3-hexenyl acetate (Frost et al., 2008). When distant parts of a plant are exposed to a HIPV signal, priming of defenses might occur. This involves expression of defense genes being primed upon receipt of a volatile signal and plants subsequently responding more vigorously to herbivore-attack than non-primed plants (Engelberth et al., 2004; Heil and Kost, 2006; Kessler et al., 2006; Heil and Silva Bueno, 2007; Frost et al., 2008). The advantage of volatile signaling is that it functions between vascularly disconnected plant parts, but also acts as a more rapid method of communication than vascular signals (Frost et al., 2007).

### HIPVs in plant to plant signalling

HIPVs that elicit defense responses within-plant may also prime or induce defenses in neighboring plants (Karban et al., 2003, 2006; Heil and Kost, 2006). This process has been observed to occur between conspecific (Karban and Shiojiri, 2009) and heterospecific individuals (Heil and Karban, 2010). Interestingly, VOCs from undamaged plants have also been shown to have an impact on the defenses of their neighbors, which indicates that it is not always the specific HIPVs that are responsible for inducing defenses (Glinwood et al., 2004, 2009). The mechanisms involved in volatile mediated plant-plant interactions have yet to be fully elucidated, although we are now seeing regular demonstrations of the complexity of the process. In sagebrush, plant-plant signaling has been shown to be more efficient between clonal cuttings of the same plant than between non-clonal conspecifics (Karban and Shiojiri, 2009; Karban et al., 2013). This indicates a degree of self or kin recognition to occur in receiver plants. It was also recently shown that hybrid aspen exposed to damaged neighbors temporally regulate two indirect defense responses, the emission of VOCs and the secretion of extra-floral nectar (EFN). EFN secretion was induced by the exposure, but not primed, whereas the emission of HIPVs was primed but not immediately induced by the exposure (Li et al., 2012), which further indicates complexity in the responses of plants to volatile signals.

The mechanisms of volatile-mediated interactions between plants require further elucidation, but there is some knowledge about the relevant signaling compounds. As for within-plant signaling, the GLVs and particularly the compound (*Z*)-3-hexenyl acetate have been implicated as providing a key signal (Kost and Heil, 2006). Other GLVs can also induce defenses in receiver plants, but in lima bean they reduce in efficiency as a signal the more they differ from (*Z*)-3-hexenyl acetate, which is the main GLV emitted by that species (Heil et al., 2008). A recent study of early responses to volatile signals by tomato receiver plants has shown that a range of volatile compounds induce depolarization of plasma membranes and cytosolic calcium flux, with green-leaf volatiles and low molecular weight molecules having a stronger impact on these responses than higher molecular weight compounds such as monoterpenes and sesquiterpenes (Zebelo et al., 2012). These early plant responses combined with the accumulating evidence in support of gene transcriptional changes in response to volatile signals could be essential in understanding the mechanisms of plant-plant signaling.

A longer exposure period and greater accumulation of HIPV compounds from damaged neighboring plants results in a greater degree of resistance against bacterial plant pathogens (Angeles Lopez et al., 2012; Giron-Calva et al., 2012) and herbivorous mites (Choh et al., 2004). This suggests that the occurrence of plant-plant signaling under natural conditions will be largely dependent on the quantity of volatiles emitted by damaged plants, the proximity of the receiver to the emitter, the sensitivity of the receiving plant and the suitability of the environment for transfer of signals. Indeed, under natural conditions the distances over which plant-plant signaling occurs are rather short, usually across distances of less than a meter (Dolch and Tscharntke, 2000; Karban et al., 2003, 2006; Heil and Adame-Álvarez, 2010). Under laboratory conditions the presence of ozone has been shown to significantly reduce the distance of signaling in lima bean (Blande et al., 2010).

### VOCs in direct defence against herbivores and pathogens

Direct defenses could affect behavior, performance or fecundity of the attacker. In many plant species specific VOCs produced by plants give them direct protection against feeding herbivores by repelling them from attacking or by deterring feeding (Egigu et al., 2011). Herbivore preference is often based on the relative proportions of constitutive volatile compounds such as the ratios of α-pinene and β-pinene (Evans et al., 1991). A whole blend of compounds that individually elicit negative responses can be attractive to aphids searching for a host plant (Webster et al., 2010). Feeding by a herbivore affects the proportions of various constitutive VOCs (e.g., Blande et al., 2007) and may thus influence the impact of HIPVs on herbivores. Specific VOCs induced by biotic stress also have specific effects on the attacking organisms, including, microbial pathogens or various herbivores. HIPVs may also have a signaling effect, whereby they repel conspecific individuals. It has been shown that female moths restrict themselves from laying eggs on plants damaged by conspecific larvae and that this decision is based on the recognition of HIPVs (De Moraes et al., 2001). Such behavior has likely evolved to hinder overcrowding, but the opposite has been observed whereby a mixture of major HIPVs is highly attractive to host seeking oligophagous moth females (Sun et al., 2012). These traits could have coevolved whereby egg induced volatiles attract herbivore females over a longer distances and indicate that suitable host plants and mating males are available in the habitat, but the final oviposition decision involves avoidance of the actual HIPV emitting plant in favor of neighbors. Utilization of foraging cues in this way could involve a number of steps such as host habitat location, host location, host recognition, host acceptance, host suitability, host (regulation and) consumption, which is similar to the classical six step framework for successful foraging by parasitoids (Vinson, 1976).

Interactions between VOCs and pathogens have not been studied extensively, but there is indication that VOCs can reduce pathogen growth. Monoterpenes (e.g., Tsao and Zhou, 2000) and GLVs (Shiojiri et al., 2006) inhibit the growth of common fungal leaf and fruit pathogens, while the sesquiterpene (*E*)-β-caryophyllene has been shown to offer *Arabidopsis thaliana* flowers a degree of defense against a bacterial pathogen (Huang et al., 2012). Exposure to the GLV (Z)-3-hexenal, resulted in significantly reduced lesions on *Botrytis cinerea* infected Arabidopsis plants (Shiojiri et al., 2006). Repellent effects of HIPVs on plant virus vectors such as aphids may result in lower infection rates and reduce the spread of aphid-transmitted plant virus diseases. However, HIPV traits can also be “hijacked” by some parasites. Cucumber mosaic virus is such a pathogen. Virus-infected plants are poor food for virus transmitting aphids, but the spread of viruses in a plant population requires feeding by virus-transmitting vector insects. HIPV emissions make infected plants attractive to two virus-vector aphid species. Brief feeding periods on poor quality plants is enough for aphids to receive the virus and then spread it onto healthy plants (Mauck et al., 2010).

### HIPVs in indirect plant defence—attraction of natural enemies of herbivores

The importance of HIPVs in attracting natural enemies of herbivores has been shown in numerous studies, mainly under laboratory conditions. There are substantial differences in composition of HIPVs from the same plant depending on the type of herbivore making the damage. For example, feeding by aphids has been shown to induce emission of methyl salicylate more distinctively than feeding by chewing herbivores (Blande et al., 2010). Feeding by the generalist feeding spider mite *Tetranychus urticae*, induced very distinctive HIPV blends from a range of different host plants (Van den Boom et al., 2002), but the predatory mite *Phytoseiulus persimilis* was still attracted to HIPVs from all of the spider mite-damaged plant species (Van den Boom et al., 2004). Generalist and specialist *Cotesia* spp. parasitoids of Brassicaceous plants seem to lack specificity at the herbivore level, whereas on the plant species level differences in HIPV attractiveness to parasitoids have been found (Geervliet et al., 1994).

## Other potential functional routes of HIPVs

**Figure 2:**
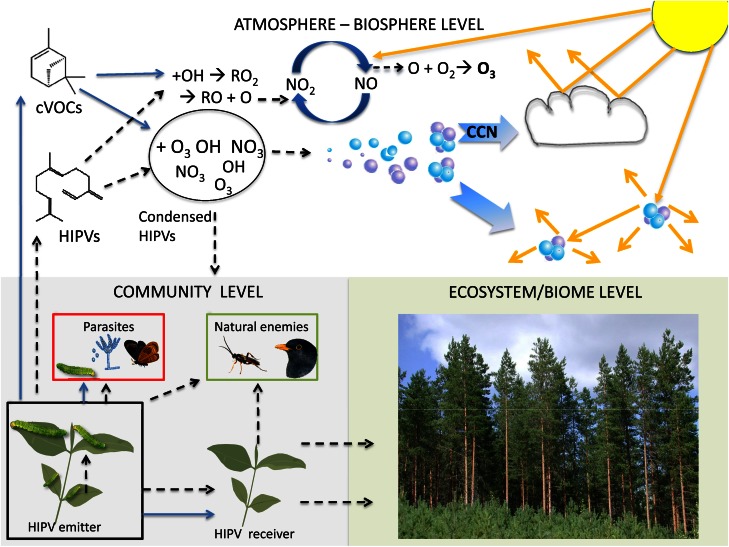
**The functions and fates of VOCs are depicted at three levels; community, ecosystem/biome and atmosphere-biosphere.** The passages of HIPVs are indicated by broken black arrows, while the passages of constitutively emitted VOCs (cVOCs) are indicated by solid blue arrows. At the community level, the functions of HIPVs include signaling from herbivore-damaged plants to plant parasites, natural enemies of the parasites and neighboring plants. Signalling within-plant via HIPVs from older leaves to younger leaves is also indicated. cVOCs are also known to be involved in host location behaviors of various plant parasites and in signaling between plants. These interactions can generally be considered as mediated by intact volatile compounds emitted by the damaged plants. After emission from plants cVOCs and HIPVs enter the atmosphere-biosphere level where they undergo various reactions that see them either re-enter the community level or have consequences on the ecosystem/biome level. In the atmosphere VOCs are influenced by ozone (O_3_), hydroxyl radical (OH) or nitrate radical (NO_3_). VOCs may lose their volatility in colder night temperatures and become sticky compounds, which may re-enter the community level as either condensed HIPVs, which adsorb to plant surfaces with effects on various community members, or as reaction products of volatiles and secondary organic aerosol (SOA) particles, which also adsorb to plant surfaces with largely unknown functions. In areas with NOx pollution the oxidation of cVOCs and HIPVs is triggered by hydroxyl radicals (OH) and results in several alkyl peroxy (RO_2_) radicals which lead to the conversion of nitric oxide (NO) to nitrogen dioxide (NO_2_). In the presence of solar radiation (yellow arrows) reactions are reversible releasing excited O atoms, which can lead to ozone formation. Ozone can then react with other VOCs in the atmosphere to form degradation products and SOA via ozonolysis. Particle growth and formation of cloud condensation nuclei (CCN) will then result in formation of cloud cover leading to enhanced albedo and reduced solar radiation at the ground level. SOA in the lower atmosphere may add diffusion of solar radiation and improve light penetration in canopies. This atmospheric interaction will feed into the ecosystem level through improving photosynthesis efficiency, but also by facilitating a net cooling effect.

The ecological functions of HIPVs described above have all been established experimentally. All rely mechanistically on the responses of a receiver organism to volatile emissions from a plant that has been subjected to a degree of stress or stimulation. While the volatile compounds remain intact they theoretically constitute a signal that can be detected and potentially perceived by organisms of the surrounding community. However, as soon as the chemicals leave the plant there are a range of potential fates or roles that could be played out. In the following section those potential adaptive roles have been classified as roles for intact HIPVs and for reaction products of HIPVs (Figure 2). In practice, in the atmosphere HIPVs and constitutively emitted VOCs cannot in most cases be separated from each other and the following description concerns the mixture of both. The following fates of VOCs and the corresponding functions are suggested:

1. Intact volatiles travel in air currents and facilitate the interactions detailed above
2. Intact volatiles, under certain environmental conditions, such as cooling temperatures, adsorb to the surfaces of surrounding vegetation including the emitting plant itself
3. Volatiles react in the atmosphere with ozone or other oxidants and thus form degradation products which could give a spatial and/or temporal dimension to the volatile signal
4. Volatiles react in the presence of NO_x_ and sunlight to produce ozone plus other degradation products, which could have lower volatility than the original VOCs
5. Degradation products of HIPVs can adsorb to plant surfaces or nucleate in the atmosphere to form secondary organic aerosol (SOA)
6. SOA particles could deposit on plant surfaces and have further ecological effects.

### Adsorption of HIPVs on neigbouring plant surfaces

It has been shown that species specific semivolatile VOCs emitted constitutively by plants can be adsorbed to the surfaces of neighboring plants (Himanen et al., 2010). There is also evidence that constitutively emitted VOCs, particularly monoterpenes, can be taken up through the stomata of neighboring plants (Noe et al., 2008; Bamberger et al., 2011). So far the ecological and ecophysiological functions of these “borrowed” VOCs are not well known, although there is evidence that the adsorbed compounds can protect receiver plants against herbivores (Himanen et al., 2010) or improve indirect defense against herbivores by attracting predators (Choh et al., 2004). Furthermore, we do not know what proportions of VOCs released by plants are adsorbed onto the surfaces of their own foliage. During the day time leaf surfaces are often warmer than the surrounding air, thus some of the semivolatile compounds will possibly ‘bounce’ between the warmer leaf surface and the colder air before condensation takes place on the colder leaf surface at night.

There is evidence that during colder night-time temperatures semi-volatile sesquiterpenoids condensate on neighboring plant surfaces. In the following morning these compounds were emitted at higher rates than any endogenous VOC compound (Himanen et al., 2010). In the afternoon the concentration of adsorbed compounds on leaf surfaces was rapidly decreased, due to evaporation from the surface as the temperature warmed. This temperature dependent behavior of sesquiterpenoids makes them an ecologically very interesting group of VOCs, because after adsorption to neighboring plant surfaces they may give protection against herbivores which attack during the late evening, night and early morning. Another important group of HIPVs, GLVs, did not show any evidence of accumulation on tested glass surfaces at +12°C or higher temperatures (Schaub et al., 2010).

### Atmospheric reactions of HIPVs and their chemical transformation

After release from the plant leaf tissue, either to be adsorbed to the leaf surface or disperse into the atmosphere, HIPVs are exposed to UV-radiation and various reactive gases which constrain their lifetimes (Kroll and Seinfeld, 2008). Therefore, the ecological functions of the original compounds synthesized by plants will only be active for a limited time, which depends on the dispersal and reactivity of the compound. There could be further potential ecological roles of HIPVs that are related to their relatively high reactivity and the properties of the rapidly formed reaction products, which include various gaseous compounds with lower volatility (Kroll and Seinfeld, 2008) and formation of solid nanoparticles (Joutsensaari et al., 2005; Virtanen et al., 2010) in secondary organic aerosols (SOA). Many of the isoprenoid oxidation products are known to be unpleasant smelling and tasting aldehydes, ketones and organic acids in gaseous or particulate form. For example, smaller SOA nanoparticles (10–20 nm) originating from α-pinene ozonolysis contain carboxylic acids, while larger particles (40 nm) have been shown to have higher concentrations of carbonyl-containing compounds and low molecular weight organic acids (Winkler et al., 2012). Earlier studies have shown that carboxylic acids and organic acids are repellent to aphids (Glinwood et al., 2003). It has also been shown that very low concentrations of aldehydes and ketones can be repellent to pollinating insects such as honey bees, (Mishra and Sihag, 2009), while the precursor monoterpenes and sesquiterpenes emitted by flowers are major floral attractants for bees (Nieuwenhuizen et al., 2009). This indicates that the functional role of plant emitted volatiles can be altered dramatically by degradation in the atmosphere. It is also known that many of the more volatile plant VOCs become less volatile in reactions with oxidants and atmospheric radicals (Kroll and Seinfeld, 2008). This may also reduce their diffusion and drift away from leaf boundary layers and increase their accumulation on the surfaces of the releasing plant.

### Formation of ozone from HIPVs and other VOCs

“Trees cause more pollution than automobiles do,” a famous quotation from USA President Ronald Reagan in 1981, indeed is partially right. When NO and NO_2_ (collectively, NOx) levels in the atmosphere are high, as occurs in environments contaminated by smoke and exhaust gases from fossil fuel combustion, VOC oxidation increases ozone levels (Lerdau and Slobodkin, 2002). Solar UV-radiation triggers the oxidation of VOCs by hydroxyl radicals (OH·), which results in several alkyl peroxy (RO_2_·) and hydroperoxy (HO_2_·) radicals. This will lead to the conversion of NO to NO_2_, and thus, promotes O_3_ accumulation and the efficient regeneration of the OH radical. Both O_3_ and OH radicals can react with other VOCs in the atmosphere (Pinto et al., 2010). In environments with cleaner air and low levels of atmospheric NOx, oxidation of biogenic VOCs removes ozone from the troposphere and promotes secondary aerosol formation by ozonolysis (Lerdau and Slobodkin, 2002; Virtanen et al., 2010). In forest environments, organic nitrates (RONO_2_) are formed via reactions of isoprene, monoterpenes and sesquiterpenes with NO_3_ radicals in the presence of OH radicals and NO. Organic nitrates are removed from the air during precipitation events and thus plant VOCs help to remove NOx from the lower troposphere (Lerdau and Slobodkin, 2002). The depositions of organic nitrogen may influence nitrogen availability from soil to vegetation and hence affect vegetation succession. On the other hand, organic nitrates could also act as atmospheric reservoirs of NOx leading to later formation of ozone and secondary organic aerosols (Pratt et al., 2012).

The ability of plants to control and even promote the formation of phytotoxic ozone in the lower troposphere may benefit plants e.g., by eliciting defense reactions which provide better plant resistance against fungal pathogens and herbivores (Sandermann et al., 1998). Cui et al. (2012) have shown that exposure of tomato plants to elevated O_3_ reduced the fecundity and prolonged the developmental time of whiteflies (*Bemisia tabaci*). Reduced performance of whiteflies was related to up-regulated pathogenesis-related protein (PR1) genes, increased phenylalanine ammonia-lyase enzyme activity and elevated concentrations of salicylic acid (SA), phenolics and condensed tannins (Cui et al., 2012). Elevated atmospheric O_3_ levels may cause slight reduction in plant growth, but plant fitness could be improved by the reduced impact of aggressive plant pathogenic fungi such as *Drechslera teres* on barley (Plessl et al., 2005). Furthermore, elevated O_3_ limits hyphal growth, sporulation and germination of conidia in many other plant pathogenic and saprophytic fungi (Tzortzakis et al., 2008; Ozkan et al., 2011).

### Secondary organic aerosols (SOA)

In the atmosphere, nano-scale aerosol particles are typically formed during the late morning and then grow throughout the day with growth rates of 1–20 nm h^−1^ (Kulmala, 2003). The smallest observed nucleating particles are 1 nm in diameter (Riipinen et al., 2012; Kulmala et al., 2013). Nucleation may be ion-induced or involve sulphuric acid or ammonia mixtures with water for the growth of nanoparticles less than 5 nm in diameter. The condensation of organic vapors in the particle size range 20–50 nm—mostly the oxidation products of plant VOCs—on particle surfaces will have increasing importance to particle size growth and several theories have been presented (Riipinen et al., 2012). The chemistry of these processes is extremely complicated. For example, an intact Scots pine seedling can emit 20 different monoterpenes (Heijari et al., 2011) and a single monoterpene of this blend, limonene, can form nearly 1200 different organic compounds in atmospheric ozonolysis reactions (Kundu et al., 2012). Finally, cloud condensation nuclei (CCN), are formed when the particle diameter reaches 30–100 nm (Riipinen et al., 2012) through addition of organic and sulphuric acid molecules.

Chamber experiments (Joutsensaari et al., 2005; Hao et al., 2009; Virtanen et al., 2010) have shown that HIPVs emitted after induction by chemical elicitors or mechanical damage can efficiently react with O_3_ and OH leading to formation and growth of atmospheric SOA particles. Insect damage to pine saplings can increase the emission rates of reactive monoterpenes and sesquiterpenes by up to 10-fold after bark feeding (Heijari et al., 2011) and up to 16-fold after needle defoliation (Ghimire et al., 2013). Recently there have been observations that in forest pest outbreak areas HIPV emissions dominate and emission rates of biogenic VOCs can increase by at least 4-fold in bark beetle outbreak areas compared to intact forests (Amin et al., 2012). This substantial increase in reactive HIPVs may significantly increase the atmospheric SOA concentrations in the affected areas (Berg et al., 2012).

Earlier studies (Mercado et al., 2009) have shown that diffusion of light by the higher particle concentrations in the atmosphere may affect photosynthesis efficiency of global vegetation. In forests the impact is caused by the better penetration of photosynthetically active light inside the tree canopy (Roderick et al., 2001). Although, there is not any direct experimental evidence that reactive HIPVs can increase light interception and improve photosynthesis through SOA formed from reactive HIPVs, this could potentially occur in insect outbreak areas. Enhanced photosynthesis rates will ultimately increase fitness of a plant, but in plant communities the emissions of an individual plant will affect the whole plant community or due to atmospheric drift of HIPVs and SOA particles, most probably conspecific plant individuals in other plant communities.

Some of the constitutively emitted plant VOCs may have much longer life times in ozone-rich atmospheres than some HIPVs that have life times of only a few minutes (Holopainen, 2011). Such compounds include e.g., isoprene (1.3 d), the monoterpenes camphene (18 d) and 1,8 cineole (110 d), and the sesquiterpene longifolene (>33d) (Atkinson and Arey, 2003). The consequence of this difference is that these constitutively emitted compounds will drift longer distances than the HIPVs compounds. Therefore, they cannot act as nucleation centers as easily as HIPV and high concentrations of HIPVs will rapidly lead to nucleation and formation of SOA particles (Joutsensaari et al., 2005; Virtanen et al., 2010), and possibly stimulate CCN formation rates locally (Riipinen et al., 2012).

The behavior of SOA particles inside a canopy and their deposition on plants and other surfaces is not yet sufficiently understood (Holopainen, 2011; Carslaw et al., 2012). Semivolatile and easily condensable HIPVs and other VOCs will condense on external plant surfaces at temperatures of +12°C and lower (Himanen et al., 2010; Schaub et al., 2010). Leaf surfaces could be a good site for ozonolysis reactions and for formation of secondary organic particles, which may also detoxify ozone before stomatal uptake Tuzet et al. (2011). The soil nitrate pool has been found to be an important source of atmospheric nitric oxide (NO) and nitrous acid (HONO) leading to atmospheric hydroxyl radical (OH) production (Su et al., 2011). This observation suggests that reactions of VOCs with OH inside the shady canopies and on leaf surfaces of smaller plants closer to the soil surface could be more common than earlier expected. These reactions may lead to formation of SOA particles (Virtanen et al., 2010; Riipinen et al., 2012) even in the boundary layer of plant leaves and stems.

## Plant evolution, HIPVs and atmospheric processes

Production of HIPVs, many of which are highly reactive in the atmosphere, is an evolved response in many plant species under biotic stress. When considering the chemical and physical properties of the HIPV compounds, a longer atmospheric life time of the compounds could possibly attract natural enemies over longer distances. However, the molecular concentration in the atmosphere will in any case be rapidly reduced due to increased distance from the point source of a damaged plant. The selection of highly reactive compounds as attractive signals in indirect plant defense does not necessarily maximize the attraction capacity of HIPVs (McFrederick et al., 2009) or improve plant fitness (Hare, 2011). Therefore, additional potential traits which can be linked to production of reactive HIPVs may give an alternative explanation for the type of HIPV compounds that evolved. Conversely, Peñuelas and Llusia (2004) proposed that an increase in biological complexity of plant physiological processes during evolution is one of the causes of the diversity of VOC emissions and their emission is just an unavoidable trait as a result of their volatility. Volk (2003) also noted that feedback loops in the biosphere contain segments based solely upon by-products of organisms' metabolisms. According to Volk (2003) these were not metabolically evolved by organisms to be sent out into the environment for altering its chemistry and do not represent a trait that was selected during evolution by natural selection.

Increased emission of reactive HIPV compounds from plants under biotic stress will inevitably lead to SOA formation as shown experimentally (Joutsensaari et al., 2005) and in a modeling study in insect outbreak areas (Berg et al., 2012). SOA particles can grow by aggregation and by VOC vapor uptake to form cloud condensation nuclei (CCN), which will have an impact on light quality reaching vegetation, influence precipitation and affect the amount of sunlight reflected to space (Riipinen et al., 2012). The importance of SOA and other aerosols in controlling global temperature and radiation balance has been convincingly shown (Arneth et al., 2009). Recently, the results from large scale measurement campaigns (Paasonen et al., 2013) suggested that VOCs emitted from vegetation substantially increase sun-screening SOA formation under climate warming. This biogenic VOC based growth mechanism produces roughly 50% of particles at the size of cloud condensation nuclei across Europe (Paasonen et al., 2013). An important question that remains to be answered, is whether HIPV have evolved to act as a part of a biosphere-atmosphere feedback system that improves the abiotic growth conditions of the plants under attack? This question prompts a further question of whether a biogenic SOA related change in environmental conditions to indirectly better defend against biotic attackers comes anywhere close to the expenditure in terms of VOCs and carbon used to create the change? If the costs of a HIPV based biosphere-atmosphere feedback system are higher than the improvement in fitness of the HIPV emitting individuals, selection of the trait does not fall in line with natural selection in a strict Darwinian sense (Moody, 2012), except perhaps in clonal plants with a population distributed across large areas (De Woody et al., 2009).

Recent evolutionary models incorporate phenotypes expressed in the external environment; however, there is still debate whether such traits generate dynamics that alter evolution (Bailey, 2012). Such models of extended phenotypes predict that the individual carrying genes for the trait, which has an impact on the physical environment, may have effects on conspecifics including an emitting individual's own offspring or siblings, but also other species (Bailey, 2012). These kinds of extended effects are particularly created by species known as ecosystem engineers (Jones et al., 1994, 2010; Hastings et al., 2007) which by niche construction modify their own niche and those of other organisms e.g., through behavior or metabolic processes.

We suggest that high HIPV emission rate capacity of Boreal conifer forest trees could be such a trait and that it may have an impact on SOA formation in insect outbreak areas (Berg et al., 2012). Higher atmospheric SOA density and associated cooling effect could provide a stabilizing feedback (Lenton, 1998; Paasonen et al., 2013) to protect conifer ecosystems against factors such as global warming, insect outbreaks and spread of invasive deciduous tree species related to warming (Kellomaki et al., 2001). It is expected that current global warming will increase the frequency of forest pest outbreaks at higher latitudes (Niemelä et al., 2001), and warmer temperatures could substantially increase the HIPV emission rates of affected conifer trees (Heijari et al., 2011; Amin et al., 2012). However, it has not yet been shown if there really is a negative feedback loop (Lenton, 1998; Lovelock, 2003) related to insect outbreak areas, i.e., the cooling effect of HIPVs through enhanced albedo by HIPV-induced SOA formation (Berg et al., 2012), CCN formation (Paasonen et al., 2013) and finally by improved cloud albedo. This cooling feedback loop would reduce the frequency of forest pest outbreaks and relieve heat stress of vegetation. More efficiently dispersed light may improve photosynthesis, but cooling would counteract the light effect, thus reducing photosynthesis rate and additionally reducing VOC production and the protective role of HIPVs against pests and pathogens.

## Where does the carbon of HIPVs go?

To return to our original question; in this review we have tried to demonstrate that the carbon fixed by a plant and then, particularly under biotic stress, released back to the atmosphere as volatile organic compounds, will have important roles in chemical, physical and biological processes during their life time. These may be facilitated by the HIPV compounds originally synthesized and emitted by a plant, or in the form of other chemical compounds after atmospheric reactions of the HIPVs. So far, we know only a fraction of the highly diverse potential routes and functions that the VOCs emitted by plants may have. For example, the carbon of highly volatile C10 monoterpenes and semivolatile C15 sesquiterpenes in the atmosphere could be bound to freshly nucleated SOA particles as a result of ozonolysis during the day time or oxidized by reactions with NO_3_ at night. The secondary organic aerosol particles formed from the same HIPV may have different chemical composition and different biological functions depending on the time of day during SOA formation. The size of SOA particles in the atmosphere may also grow by adsorption of other organic vapors to their surface (Kroll and Seinfeld, 2008) or the particles could be influenced by solar UV irradiation leading to the photolysis and formation of several oxygenated C1–C3 compounds (Pan et al., 2009). The reactions could also be reversible and low molecular mass carbon compounds may react again to form larger carbon-based molecules. Finally the carbon bound to the HIPV compounds will be oxidized to CO or CO_2_ in the atmosphere (Kroll and Seinfeld, 2008) and may again be utilized by plants in the process of photosynthesis, while it could alternatively end up as organic polymers in the sediments of terrestrial or aquatic ecosystems.

### Conflict of interest statement

The authors declare that the research was conducted in the absence of any commercial or financial relationships that could be construed as a potential conflict of interest.
